# Kidney Diseases

**Published:** 1895-04-20

**Authors:** 


### Progress in Medicine.

### KIDNEY DISEASES.

Sir George Johnson[1] criticises the theory of "renal inadequacy," pointing out that if it existed we should find urea and other products accumulated in the blood, with high arterial tension. He thinks that what really occurs is defective assimilation and oxidation, just as in cholera collapse. Here suppression of urine and of bile arises from deficiency of oxidation in the system, while the secretion of milk, which does not depend on oxidation, continues. Deficiency of urea and failure to recover from injuries depends not on inadequacy of the kidneys, but on failure of digestion and nutrition.

The pathology of arterio-sclerosis is discussed by Weber, who holds it doubtful whether the change begins in the tunica externa or intima, and whether it is of inflammatory nature at all. The numerous alleged causes are examined, and shown to be insufficient or unproved in a paper of valuable though destructive criticism.

The etiology of Bright's disease is reviewed by M'Lachlan[2], who concludes that all things which tend to weaken the general health, which throw too much work on the kidney, and which disturb the efflux of blood from it are predisposing causes, while the exciting ones are those poisons from fevers, dyspepsia, and other sources which have to be eliminated by it. The most constant indication of the presence of these impurities is hyperacidity of the urine. Two cases of nephritis lately occurred from the inunction of B. naphthol for scabies.[3] A 2 per cent. ointment had been used, and albuminuria, œdema, and blood casts followed, one of the patients dying. The kidneys were engorged, and cloudy swelling of the convoluted tubules was present. The action of ether on the kidneys has been recently tested by Wood[4], who finds that congestion of the kidney with cloudy swelling in the convoluted tubules takes place, and probably desquamation, if the irritation is repeated. Little or no ether is excreted by these organs, and sudden death may occur when ether is given in the uræmic state.

An interesting discussion has arisen as to the action of asparagus on the kidney.[5] Wilks wrote that he had found it was inhibitory, instead of being, as commonly stated, a diuretic. This view was confirmed by three other writers. Ralfe[6] noted that on eating white asparagus there was increased frequency of micturition, with diminution in quantity of one-third. The peculiar principle of asparagus appears to be found chiefly in the white part, and is probably methyl mercaptan. (The latter substance, together with $H_2S$, was found by Marklus lately in the urine of a convalescent from pneumonia,[7] and ascribed to the action of a bacillus which he cultivated on agar). On the other hand, B. Yeo, using apparently the green portion, found an increase of 10 to 20 ounces of urine daily,[8] and insists that asparagin has diuretic properties. Similar discussions have taken place as to the action of diuretin. McPhedran[9] finds it useful in only early stages of heart failure with disease of the kidney, less so in purely renal cases, but the action is temporary, and may be accompanied with depression and headache. Lactose was tested in 44 cases, and found by Ryerson[10] much more efficient than when given in its natural combination in milk as a remedy for chronic Bright's disease. He claims for it uniform and permanent improvement with rapid action. He gives from 20 to 50 grains three times daily. The action of strontium salts is reviewed by Da Costa,[11] who finds them valuable in acute forms, but less so in chronic Bright's diseases. They are admirable diuretics, and slightly reduce the amount of albumen, especially in acute cases. Where a pure milk diet is not tolerated legumen is recommended by Boret in daily doses of 1½ ounces dissolved in milk or other fluids.[12] It is very easily digestible and nourishing. The action of piperazine upon existing calculi is denied by Bohland, and though many reports of its value in gout have been published, he insists that we are quite in the dark as to the means by which this may be brought about. The quantity of uric acid daily excreted remains unaltered under piperazine, but the urine is perhaps less acid. However, as we noticed previously, Stewart, in view of the clinical improvement witnessed, suggests that the uric acid may be really transformed into urea, and thus escape detection. Gordon Sharp finds that it is not wholly oxidised in the body, but may be detected in the urine.[13] One per cent. solutions in normal urine at the body heat dissolve to a great extent uric acid calculi. The undissolved portion is converted into a soft granular state. Great relief from pain and general improvement under piperazine is recorded by S. W. C. Brown in a case of nephritic colic.[14] McKinlock reports[15] four severe cases of it in which he gave five or even ten-grain doses with an equal amount of phenocoll every two hours, with rapid disappearance of pain, increased diuresis, and the passing of quantities of uric acid fragments. He noted no untoward symptoms except some slight twitching of the limbs in the case where he gave 3¼ drachms in 48 hours. In an instructive paper on the same disease, benzoate of lithium, 20 grains daily, is recommended by L'Union Médicale, with large quantities of Vals or Contrexeville water at bedtime.[16] The writer also insists upon careful dieting, a teaspoonful of Rochelle salt in the morning, and alkalies if necessary. For oxaluria 15 grains of phosphate of soda should be taken after meals and at bedtime, and often a diet of milk and eggs will be found of use.

Sir G. Johnson writes on substances mistaken for sugar in the urine, viz., uric acid and kreatinine, which reduce copper solutions.[17] Three-fourths of the reduction in normal urine is effected by kreatinine, and the remainder by uric acid. He concludes that there is an entire absence of sugar in the healthy secretion, as shown by the researches of G. S. Johnson. On picric acid only kreatinine possesses this reducing power, and if on testing urine with picric acid and liq. potassæ at normal temperatures a red colour appears kreatinine is present, and if afterwards on boiling a bright red light is transmitted through the tube there is, he believes, no sugar present, but only kreatinine. If no reduction occurs at normal temperatures, but only on boiling, sugar is the agent. Salicylates also redden picric acid and slightly reduce copper through their decomposition products.

His proof that normal urine contains no sugar depends on: (1) The phenyl hydrazene test, and (2) the fact that if uric acid and kreatinine are removed there is no reducing power left.

[1] Brit. Med. Journ., July 28th. [2] Glasgow Med. Journ., Oct. [3] Brit. Med. Journ., Oct. 6th. [4] New York Med. Journ., Sept. 22nd. [5] Ther. Gazette, Sept. [6] Lancet, June 30th. [7] Forts. d. Med. Journ. [8] Lancet, June 3rd. [9] Canadian Pract., June. [10] Med. Record, August 4th. [11] Ther. Gazette, Ju'y 16th. [12] Amer. Journ. Med. Sci., Oct. [13] Ther. Gazette, Sept. 15th. [14] Therapist, July 14th. [15] New York Med. Journ., August 18th. [16] Ther. Gazette, Sept. 16th. [17] Lancet, July 7th

